# Supplementary material for: Burden of COVID-19 Pandemic Perceived by Polish Patients with Multiple Sclerosis
Source: J Clin Med. 2021 Sep 17;10(18):4215. doi: 10.3390/jcm10184215 (PMC8468201; doi:10.3390/jcm10184215)
Supplement: Supplementary file 1 [file jcm-10-04215-s001.zip › jcm-1390075-supplementary.pdf]

Supplementary Materials: Covid MS questionnaire.

Date of birth:

Sex: female

male

Marital status: Single

Stable relationship

Married

Divorced

Widowed

Children : YES/NO ; If YES – how many

Place of residence: big city (> 100 000 inhabitants)

Small town (< 100 000 inhabitants)

Village

Level of education: primary school

Vocational school

High school diploma

University degree

Occupational status: employed (full-time)

Employed (part-time)

Self-employed

Unemployed

Retired

Disability pensioner

Student

II. MS-related items:

1. When did the first symptoms of MS occur? (year)
2. When were you diagnosed with MS? (year)
3. What type of MS do you have:

Relapsing-remitting

Secondary or primary progressive

I do not know

4. Which of the MS symptoms listed below are the most debilitating for you (please mark up to 3):

- Disturbed gait
- Weakness or discoordination of upper limbs
- Visual deficit
- Vertigo/balance problems
- Sensory impairment (sensory loss, tingling/burning sensations, pain)
- Bowel and/or bladder problems
- Fatigue
- Cognitive impairment (problems with memory, attention etc)
- Other, please specify……………………………………………

5. Please choose ONE from the following options, which best describes your mobility:

- I can walk any distance without rest
- I can walk a limited distance, then I have to rest
- I can only walk using an assistive device (cane, crutch, walker etc) or with accompanying person
- I use a wheelchair

6. What kind of treatment do you receive because of MS:

- disease-modifying treatment (DMT) within therapeutic program
- experimental therapy within clinical trial
- other treatment

7. Please provide the name(s) of your MS medications and how long you receive them

……..

……..

8. Apart from MS, do you have any other diseases recognized? YES/NO

If YES, please specify

………..

III. MS course during the pandemic (from March 2020 until now):

1. Have you experienced a relapse of MS? YES/NO

If YES,

- Was it confirmed by a neurologist? YES/NO
- Were you admitted to hospital? YES/NO
- Were you treated with infusions of corticosteroids? YES/NO
- Were you treated with oral corticosteroids? YES/NO

2. Were your most debilitating MS symptoms more frequent or more severe? YES/NO

If YES, which of the following ones?

- Disturbed gait
- Weakness or discoordination of upper limbs
- Visual deficit
- Vertigo/balance problems
- Sensory impairment (sensory loss, tingling/burning sensations, pain)
- Bowel and/or bladder problems
- Fatigue
- Cognitive impairment (problems with memory, attention etc)

3. Have you experienced any new symptoms (other than listed above) YES/NO

If YES, please specify…………………………………………..

4. Have you had any problems
   - with access to a neurologist/MS care center? YES/NO
   - With access to a primary care physician or other specialist? YES/NO
   - With access to rehabilitation? YES/NO
   - With access to any relevant information concerning MS and pandemic? YES/NO

5. Because of pandemic, have you had any problems:
   - With continuation of treatment? YES/NO
   - With starting DMT? YES/NO
   - With switch of DMT? YES/NO

6. Have you cancelled any scheduled visit to the hospital or MS center because of fear of infection? YES/NO

If YES, were you offered remote consultation (tele-consultation, phone call etc)?

7. During the pandemic, did you need any additional help or care? YES/NO

IV. Exposure to COVID-19 (since March 2020 till now)

1. Were you tested for COVID-19? YES/NO
2. Were you found positive for COVID-19? YES/NO

   If YES, were you admitted to hospital? YES/NO

3. Did you undergo a quarantine?
4. Was any of your family or friends found positive for COVID-19? YES/NO

V. Other consequences of pandemic

During the pandemic (since March 2020 till now):

1. Have you experienced any work-related problems? YES/NO

If YES, please choose all that apply:

- Limited or suspended schedule of work
- Loss of job
- Problems with remote work/studying
- Fear from infection at workplace
- Increased range of responsibilities

2. Have you experiened any financial problems? YES/NO
3. Have you experienced any problems associated with family/close persons? YES/NO

If YES, please choose all that apply:

- COVID-19 infection in close person
- Concern about health of close persons
- Need to provide additional care/help for close person
- Helping children with e-learning
- Organizing remote work and studying for all the family members
- Other conflict situations associated with staying at home during lockdown
- Limited contacts with close persons

4. Have you experienced any problems with your daily activities? YES/NO

If YES, please choose all that apply:

- Shopping
- Household duties (cleaning etc)
- Small repairs
- Pet care
- Other, please specify…………………………………………..

5. Because of pandemic, did you have to cancel or postpone any important life event (travel, celebration etc)? YES/NO
